# BL153 Partially Prevents High-Fat Diet Induced Liver Damage Probably via Inhibition of Lipid Accumulation, Inflammation, and Oxidative Stress

**DOI:** 10.1155/2014/674690

**Published:** 2014-04-03

**Authors:** Jian Wang, Chi Zhang, Zhiguo Zhang, Qiang Chen, Xuemian Lu, Minglong Shao, Liangmiao Chen, Hong Yang, Fangfang Zhang, Peng Cheng, Yi Tan, Ki-Soo Kim, Ki Ho Kim, Bochu Wang, Young Heui Kim

**Affiliations:** ^1^College of Bioengineering, Chongqing University, Chongqing 400044, China; ^2^The Chinese-American Research Institute for Diabetic Complications, School of Pharmaceutical Sciences & Key Laboratory of Biotechnology Pharmaceutical Engineering, Wenzhou Medical University, Wenzhou 325035, China; ^3^Department of Pediatrics of the University of Louisville, The Kosair Children's Hospital Research Institute, Louisville, KY 20202, USA; ^4^Rui'an Center of the Chinese-American Research Institute for Diabetic Complications, The Third Affiliated Hospital of the Wenzhou Medical University, Wenzhou 325200, China; ^5^Department of Cardiology at the First Hospital & School of Public Health, Jilin University, Changchun 130021, China; ^6^Bioland Biotec Co., Ltd., Zhangjiang Modern Medical Device Park, Pudong, Shanghai 201201, China; ^7^Bioland R&D Center, 59 Songjeongni 2-gil, Byeongcheon, Dongnam, Cheonan, Chungnam 330-863, Republic of Korea

## Abstract

The present study was to investigate whether a * magnolia* extract, named BL153, can prevent obesity-induced liver damage and identify the possible protective mechanism. To this end, obese mice were induced by feeding with high fat diet (HFD, 60% kcal as fat) and the age-matched control mice were fed with control diet (10% kcal as fat) for 6 months. Simultaneously these mice were treated with or without BL153 daily at 3 dose levels (2.5, 5, and 10 mg/kg) by gavage. HFD feeding significantly increased the body weight and the liver weight. Administration of BL153 significantly reduced the liver weight but without effects on body weight. As a critical step of the development of NAFLD, hepatic fibrosis was induced in the mice fed with HFD, shown by upregulating the expression of connective tissue growth factor and transforming growth factor beta 1, which were significantly attenuated by BL153 in a dose-dependent manner. Mechanism study revealed that BL153 significantly suppressed HFD induced hepatic lipid accumulation and oxidative stress and slightly prevented liver inflammation. These results suggest that HFD induced fibrosis in the liver can be prevented partially by BL153, probably due to reduction of hepatic lipid accumulation, inflammation and oxidative stress.

## 1. Introduction

Obesity is becoming a health issue all over the world. It grows rapidly and always leads to severe complications such as cardiovascular disorder, diabetes, and cancer [1–3]. Liver is one of the most affected organs by obesity in the body, which leads to nonalcoholic fatty liver disease (NAFLD) [4]. NAFLD is a pathologic entity, including a spectrum of liver damage ranging from simple steatosis to nonalcoholic steatohepatitis (NASH), advanced fibrosis, and progression to cirrhosis [5]. The pathogenesis of NAFLD appears to involve a 2-hit process [6–8]. The first hit is the steatosis which is believed to be triggered by insulin resistance and the second hit which involves oxidative stress results in disease progression. The proinflammatory cytokines have been implicated in the pathogenesis of NAFLD and contribute to the increased risk for hepatocellular carcinoma. Therefore, attenuation of lipid accumulation, oxidative damage, and inflammation associated with obesity is expected to exert beneficial effects and thus be a potential novel therapeutic strategy for NAFLD.

*Magnolia officinalis* is regarded as Chinese traditional medicine and used in the clinical practice for a long time to treat various disorders [9, 10]. Several constituents of the* Magnolia* such as honokiol, obovatol, and magnolol have been reported to have antioxidative [11, 12] and anti-inflammatory effects [13–15]. Honokiol has been shown to have the effect of anti-inflammation by inhibiting NF-*κ*B activation, activin phosphorylation, and subsequent I*κ*B*α* degradation [16, 17]. Consistent with the suppression effect of honokiol on NF-*κ*B is that honokiol decreases levels of NF-*κ*B target genes including tumor necrosis factor (TNF-) *α*, intercellular adhesion molecule (ICAM-) 1, and plasminogen activator inhibitor (PAI-) 1. Honokiol also plays critical role in scavenging reactive oxygen species via inhibition of TNF-*α* mediated NADPH oxidase (NOX) pathway in hepatocytes [18]. In addition to honokiol, other two main constituents of* Magnolia*, magnolol, and obovatol also showed the antioxidative effect by attenuation of ROS generation and the subsequent reduction of NF-*κ*B activation [19, 20]. Both oxidative stress and inflammation cause fibrosis, which can be prevented by attenuation of transforming growth factor beta 1 (TGF-*β*1) and its downstream profibrotic factors such as connective tissue growth factor (CTGF) through inhibition of Smad-2/3 signaling pathway [21]. Furthermore, it was reported that magnolol regulated lipid metabolism by increase of fatty acid *β*-oxidation and lipolysis, finally reducing lipid accumulation in the tissue [22, 23].

The present study was to clarify whether BL153 has protective effects on HFD-induced liver damage and if so what is the possible mechanism. We found administration of BL153 significantly prevented chronic obesity-induced liver damage with the mechanism of reducing lipid accumulation and inhibiting inflammation and the associated oxidative stress.

## 2. Material and Methods

### 2.1. Experimental Protocols and Animals

*Magnolia* extract (BL153) was prepared as our previous report [24]. The major constituents and their structure of* magnolia* extract have been defined in previous studies [24–26]. All experiments involving mice conformed to the National Institutes of Health Guide for the Care and Use of Laboratory Animals and were approved by the University of Louisville Institutional Animal Care and Use Committee. Male C57BL/6J mice at 8 weeks of age were purchased from the Jackson Laboratory and housed in the University of Louisville Research Resources Center at 22°C with a 12-hour light/dark cycle. Mice were randomly divided into five groups (*n* = 5) and fed either a control diet (Ctrl, 10% kcal as fat; D12450B, Research Diets Inc. NJ) or a high fat diet (HFD, 60% kcal as fat; D12492B, Research Diets Inc. NJ) with or without BL153 for six months: (1) Ctrl group: mice were fed a control diet and supplemented with 0.5% ethanol; (2) HFD group: mice were fed a HFD and supplemented with 0.5% ethanol; (3) HFD+2.5 mg/kg group: mice were fed a HFD and supplemented with BL153 at the dose of 2.5 mg/kg; (4) HFD+5 mg/kg group: mice were fed a HFD and supplemented with BL153 at the dose of 5 mg/kg; and (5) HFD+10 mg/kg group: mice were fed a HFD and supplemented with BL153 at the dose of 10 mg/kg. Selection of 5 mg/kg and 10 mg/kg for the present study was based on a previous study [25], where treatment with BL153 at these two dose levels for a week showed a significantly protective effect. Since the treatment in the present study is longer than that, we also included one lower dose of BL153 at 2.5 mg/kg.

For preparing BL153 gavage solution, different doses of BL153 were dissolved into 100% ethanol and then diluted with ddH_2_O into final concentration of 1.0 mg/mL (high dose group), 0.5 mg/mL (middle dose group), and 0.25 mg/mL (low dose group) with final concentration of ethanol at 0.5%, respectively. Therefore, the gavage volume was 1% (mL/g) of mouse body weight (e.g., 25 g mouse should be given 250 µL). Control groups were given same volume of ddH_2_O with 0.5% ethanol. During the six-month feeding, body weight was measured every month, and the gavage volume was justified based on the body weight change. At the end of experiment, all mice were sacrificed and livers were collected for further analysis.

### 2.2. Histological Examination and Immunohistochemical Staining

The fixed liver tissue was cut into 3 mm thickness blocks. The tissue blocks were embedded in paraffin and cut into 4 µm slices. After being deparaffinized using xylene and ethanol dilutions and rehydration, the sections were stained with hematoxylin and eosin (H & E, DAKO, Carpinteria, CA) to examine the tissue structure, inflammatory cell infiltration, necrosis, and lipid accumulation as described previously [27, 28]. For immunohistochemical staining, sections were blocked with Superblock buffer (Pierce, Rockford, IL) for 30 min. Sections were then incubated with proper primary antibodies in 1 : 200 dilutions overnight at 4°C. After three washes with phosphate-buffered saline (PBS), these sections were incubated with horseradish peroxidase-labeled secondary antibody (Santa Cruz Biotechnology, Santa Cruz, CA) at room temperature for 1 h, followed by color development with diaminobenzidine for 2 min.

### 2.3. Oil Red O Staining and Triglyceride Assay for Lipid Accumulation

Cryosections from OCT-embedded tissue samples of the liver (10 mm thickness) were fixed in 10% buffered formalin for 5 min at room temperature, stained with Oil Red O for 1 h, washed with 10% isopropanol, and then counterstained with hematoxylin for 30 s. A Nikon microscope (Nikon, Melville, NY) was used to capture the Oil Red O-stained tissue sections at 40x magnification. For hepatic triglyceride levels assay, 200 mg of hepatic tissues was homogenized at 4°C in 2.0 mL diluted Standard Diluent using a Polytron tissue homogenizer. After centrifugation at 10000 ×g for 10 min at 4°C, samples were diluted by the ratio of 1 : 5 using the diluted Standard Diluent. Then, the triglyceride levels in liver tissue were measured according to the manufacturers' instructions provided in the triglyceride colorimetric assay kit (Cayman Chemical, CA).

### 2.4. Western Blot

Western blot assays were performed as described before [29]. Briefly, liver tissues were homogenized in RIPA lysis buffer (Santa Cruz Biotechnology, Santa Cruz, CA). Proteins were collected by centrifuging at 12,000 g at 4°C. The sample of total protein was separated on 10% SDS-PAGE gels and transferred to nitrocellulose membranes (Bio-Rad, Hercules, CA). These membranes were rinsed briefly in tris-buffered saline containing 0.05% Tween 20 (TBST), blocked in blocking buffer (5% milk and 0.5% BSA) for 1 h, and then incubated with different primary antibodies overnight at 4°C, followed by three washes with TBST and incubation with secondary horseradish peroxidase-conjugated antibody for 1 h at room temperature. Antigen-antibody complexes were then visualized using ECL kit (Amersham, Piscataway, NJ). The primary antibodies used here include those against 3-nitrotyrosine (3-NT, 1 : 2000, Millipore, Billerica, MA), 4-hydroxynonenal (4-HNE, 1 : 2000, Alpha Diagnostic International, San Antonio, TX), ICAM-1 (1 : 500), CTGF and *β*-actin (1 : 1000, Santa Cruz Biotechnology, Santa Cruz, CA), PAI-1 (1 : 2000, BD Biosciences, Sparks, MD), TNF-a (1 : 500), and TGF-*β*1 (1 : 1000; Cell Signaling, Danvers, MA).

### 2.5. Statistical Analysis

Data were collected from five animals for each group and presented as mean ± SD. One-way ANOVA was used to determine general difference, followed by a post hoc Tukey's test for the difference between groups using Origin 7.5 laboratory data analysis and graphing software. Statistical significance was considered as *P* < 0.05.

## 3. Results

### 3.1. HFD-Induced Obesity and the Effects of BL153

**Figure 1:**
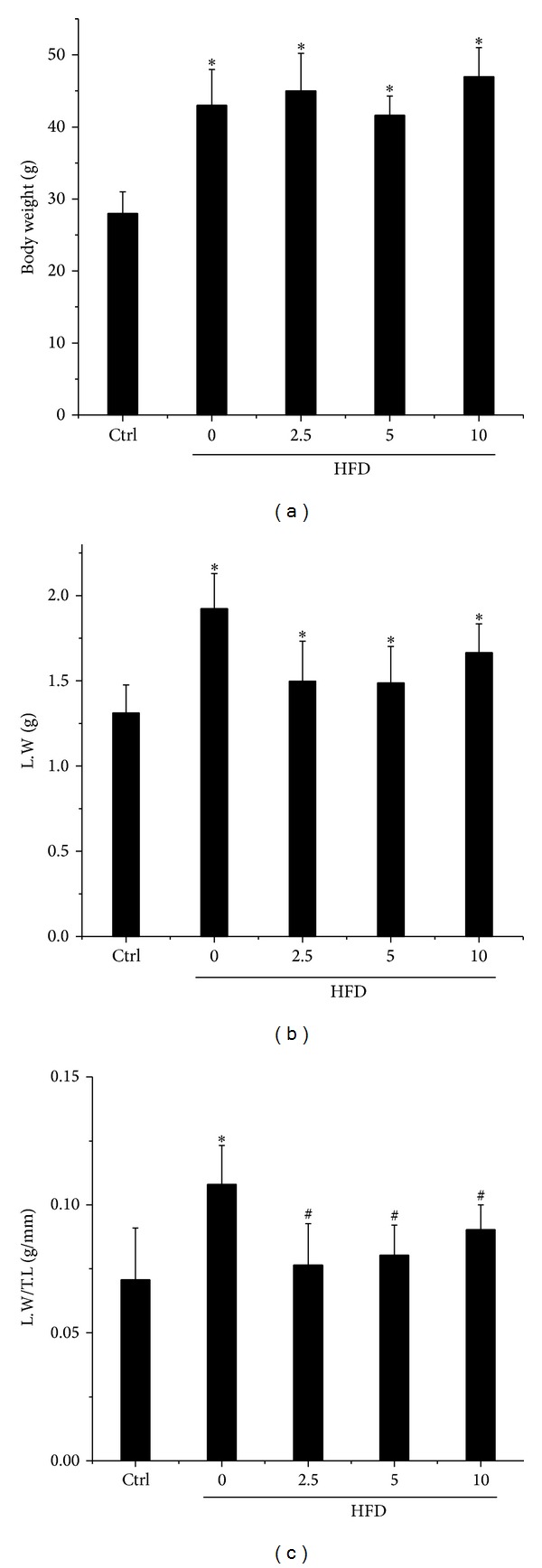
Effects of BL153 on body weight, liver weight, and the ratio of liver weight to tibia length. Mice were fed HFD to induce obesity; mouse models were simultaneously treated with or without BL153 at three dose levels (2.5, 5 or 10 mg/kg body weight) by gavage. The body weight (a) was monitored at 6 months after HFD feeding. Then, the mice were sacrificed and the liver weight (b) and the ratio of liver weight to tibia length (c) were examined. Data were presented as means ± SD (*n* = 5 at least in each group). *P < 0.05 versus Ctrl group. ^#^ *P* < 0.05 versus HFD group. L.W = liver weight; T.L = tibia length; Ctrl: control; HFD: high fat diet.

In order to identify whether BL153 can prevent obesity and the subsequent hepatic injury, HFD treatment was applied in this study to induce obesity mouse model. After 6 months of HFD feeding, the body weight was significantly increased, which indicated the establishment of the obesity mouse model (Figure 1(a)) but treatment with BL153 had no significant effects on HFD-induced body weight gain (Figure 1(a)). Additionally, HFD also significantly increased liver weight (Figure 1(b)) and the ratio of liver weight to tibia length (Figure 1(c)); treatment with BL153 slightly prevented HFD-induced liver weight increase (Figure 1(b)) but significantly prevented the ratio of liver weight to tibia length (Figure 1(c)), a more reasonable indicator of liver hypotrophy.

### 3.2. BL153 Attenuated HFD-Induced Hepatic Fibrosis

**Figure 2:**
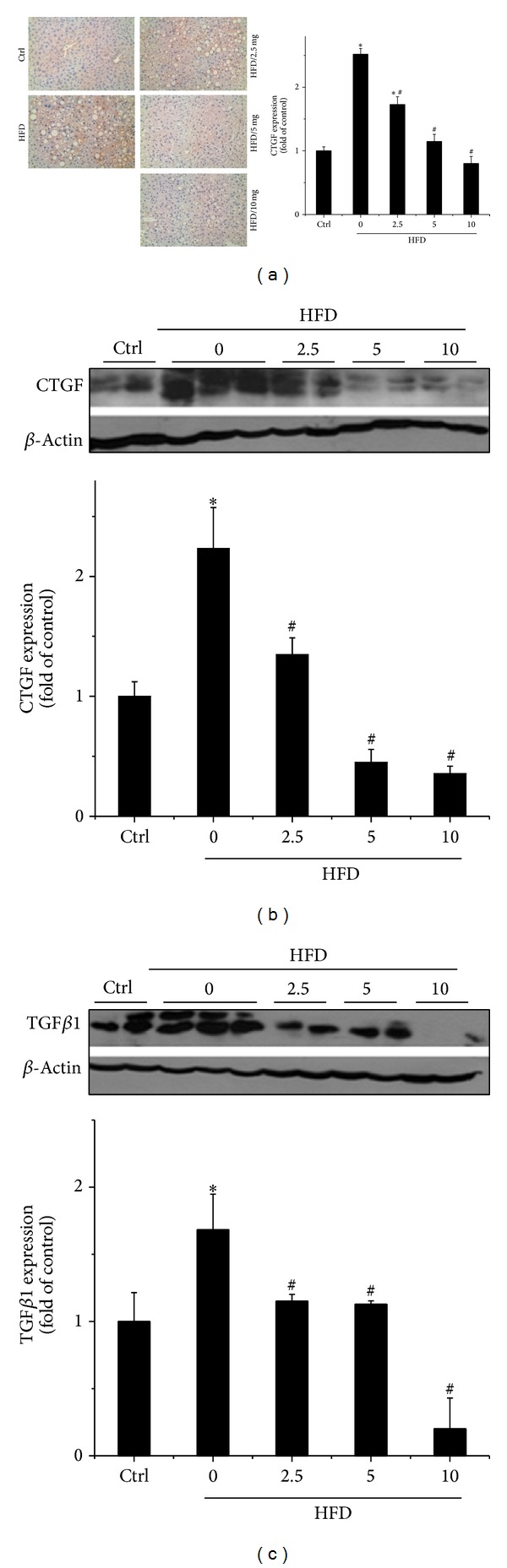
Effects of BL153 on HFD-induced fibrosis in the liver. The expression of fibrotic molecular maker CTGF was detected by immunohistochemical staining (a), and both CTGF (b) and TGF-*β*1 (c) were also detected by Western blot. Data were presented as means ± SD (*n* = 5 at least in each group). *P < 0.05 versus Ctrl group. ^#^ *P* < 0.05 versus HFD group. CTGF: connective tissue growth factor; TGF-*β*1: transforming growth factor *β*1; Ctrl: control; HFD: high fat diet.

Liver weight increase is a feature of hepatic hypertrophy which is closely associated with liver fibrosis [30–32]. Moreover, fibrosis is a key step of the development of NAFLD [33, 34]. We next identified whether administration of BL153 prevents hepatic fibrosis under obese conditions. Western blot assay and immunohistochemical staining revealed that HFD treatment significantly upregulated hepatic CTGF expression which was significantly attenuated by administration of BL153 in a dose-dependent manner (Figures 2(a) and 2(b)). In order to further confirm our findings about the antifibrotic effect of BL153 in HFD fed mice, we also examined the expression of another classical fibrotic marker TGF-*β*1. Similar protective effects were observed that HFD significantly increased hepatic TGF-*β*1 expression, which was remarkably reduced by treatment of BL153 in a dose-dependent manner (Figure 2(c)).

### 3.3. Effects of BL153 on HFD-Induced Hepatic Steatosis

**Figure 3:**
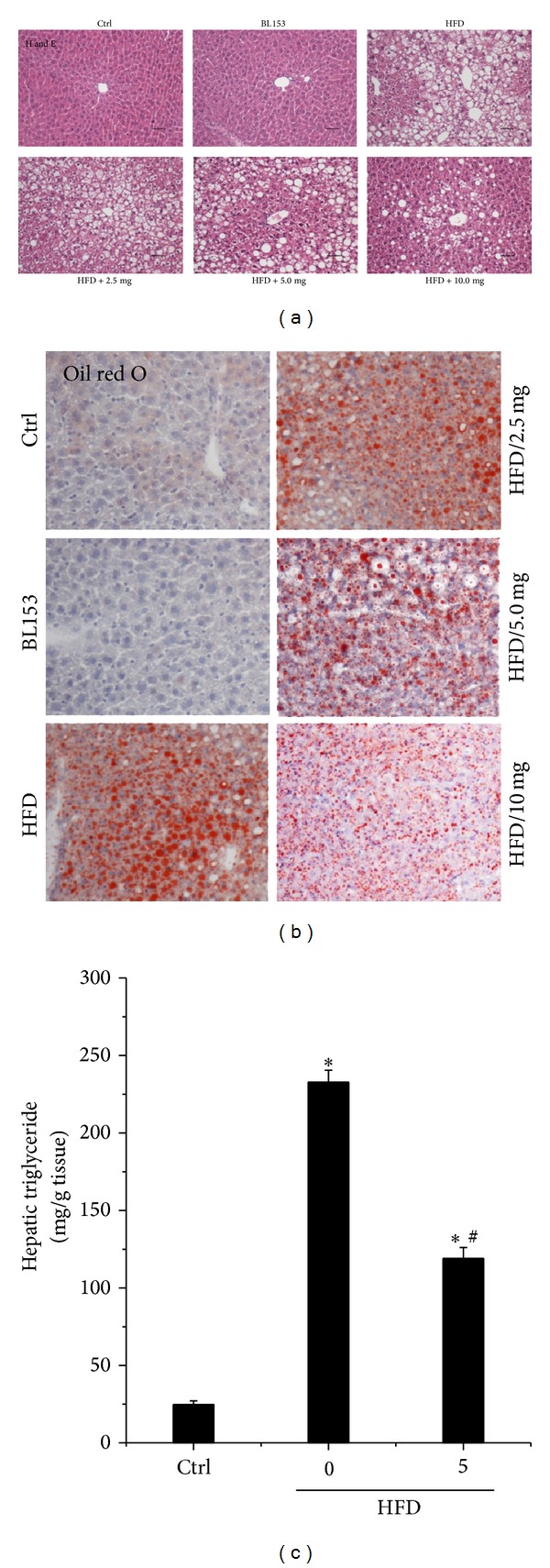
Effects of BL153 on HFD-induced hepatic lipid accumulation. Hepatic morphological changes were examined microscopically with H&E staining ((a) original magnification = 40). Hepatic lipid accumulation was examined by Oil Red O staining ((b) original magnification = 40) and triglyceride level assay (c). Data were presented as means ± SD (*n* = 5 at least in each group). *P < 0.05 versus Ctrl group; ^#^ *P* < 0.05 versus HFD group. Ctrl: control; HFD: high fat diet.

The above study revealed that BL153 significantly prevented HFD-induced hepatic hypertrophy and fibrosis. And lipid accumulation is the first step of NAFLD development [35, 36]. Thus, we tried to determine whether BL153 can prevent HFD-induced hepatic steatosis. Liver pathological examination with H&E staining is presented in Figure 3(a). The hepatic cell structure in Ctrl group was normal. However, HFD feeding increased hepatic damage with obviously hepatic necrosis (Figure 3(a)). Further examination of hepatic lipid accumulation status with Oil red O staining and triglyceride level assay revealed that HFD feeding significantly induced hepatic lipid accumulation compared to Ctrl group (Figures 3(b) and 3(c)). Administration of BL153 significantly, but not completely, prevented HFD-induced hepatic lipid accumulation (Figures 3(b) and 3(c)).

### 3.4. BL153 Attenuated HFD-Induced Hepatic Inflammation

**Figure 4:**
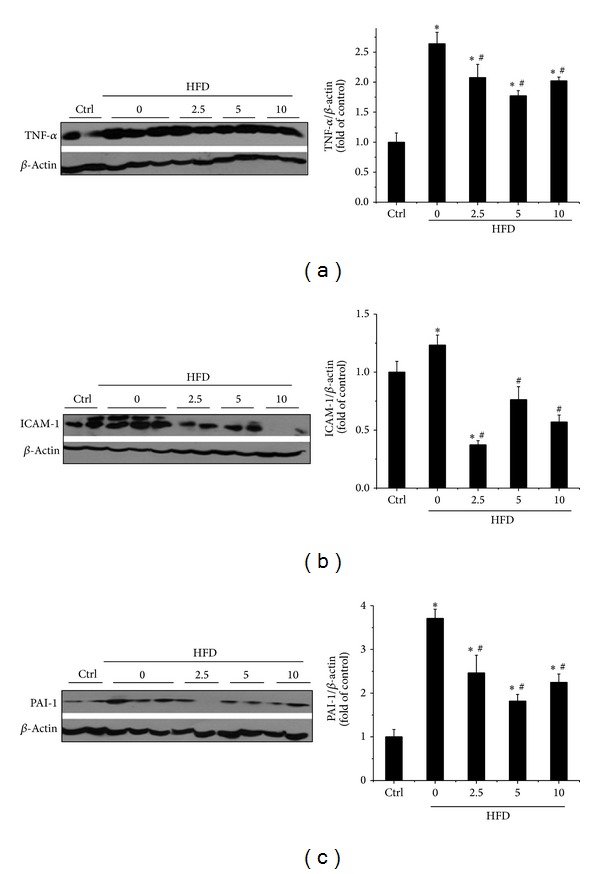
Effects of BL153 on HFD-induced hepatic inflammation. The expression of inflammatory factors, including TNF-a (a), ICAM-1 (b), and PAI-1 (c) was examined by Western blot. Data were presented as mean ± SD (*n* = 5 at least in each group). *P < 0.05 versus Ctrl group; ^#^ *P* < 0.05 versus HFD group. TNF-a: tumor necrosis factor a; ICAM-1: intercellular adhesion molecule-1; PAI-1: plasminogen activator inhibitor-1; Ctrl: control; HFD: high fat diet.

Inflammation is the main pathological consequence of HFD-induced obesity characterized by release of inflammatory factors which contributes to insulin resistance [37–39]. Thus, we determined whether BL153 can prevent HFD-induced hepatic inflammation. The protein expression of classic inflammatory factors including ICAM-1, TNF-*α*, and PAI-1 was detected. Western blot assay revealed that HFD significantly upregulated the expression of TNF-*α* (Figure 4(a)), ICAM-1 (Figure 4(b)), and PAI-1 (Figure 4(c)) in the liver. However, all three doses of BL153 treatment significantly attenuated HFD-induced upregulation of TNF-*α* (Figure 4(a)), ICAM-1 (Figure 4(b)), and PAI-1 (Figure 4(c)), while no significant differences among the three doses of BL153 treatment were observed.

### 3.5. BL153 Attenuated HFD-Induced Hepatic Oxidative Stress

**Figure 5:**
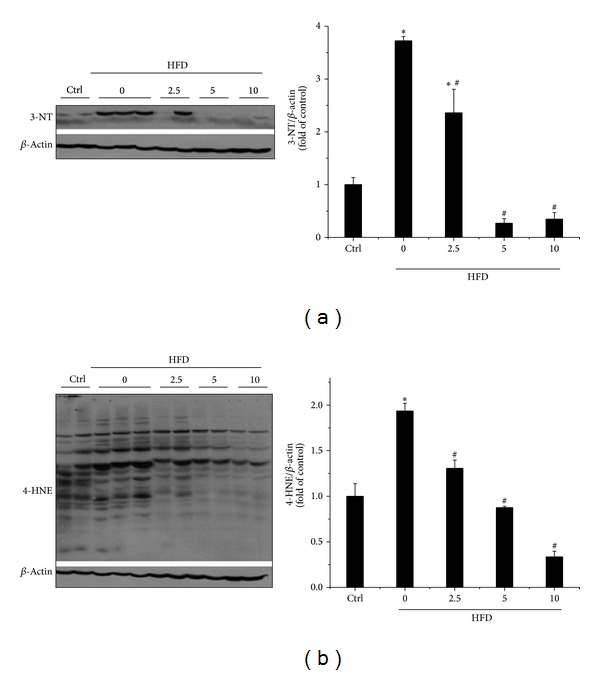
Effects of BL153 on HFD-induced hepatic oxidative stress. Hepatic expression of oxidative stress marker 3-NT (a) and 4-HNE (b) was examined by Western blot. Data were presented as mean ± SD (*n* = 5 at least in each group). *P < 0.05 versus Ctrl group; ^#^ *P* < 0.05 versus 3-NT: 3-nitrotyrosine; 4-HNE: 4-hydroxynonenal HFD group. Ctrl: control; HFD: high fat diet.

HFD-induced obesity generally leads to oxidative stress via release of multiple adipokines which in turn generates excessive reactive oxygen species [40]. Furthermore, obesity-associated inflammation is an oxidative stress enhancer, which interacts with each other and causes a vicious circle, promoting the development of insulin resistance [41, 42]. Thus, we next determined whether BL153 can prevent HFD-induced oxidative stress measured by 3-NT as an index of nitrosative damage (Figure 5(a)) and 4-HNE (lipid peroxide) as an index of oxidative damage (Figure 5(b)). The result showed that HFD feeding significantly upregulated the expression of 3-NT and 4-HNE in the liver, which were significantly attenuated by BL153 treatment in a dose-dependent manner (Figures 5(a) and 5(b)).

## 4. Discussion

Obesity is currently a worldwide epidemic and among the most challenging health conditions. A major metabolic consequence of obesity is insulin resistance which underlies the pathogenesis of the metabolic syndrome such as NAFLD, the hepatic manifestation of obesity and metabolic syndrome [6, 43]. NAFLD is considered to be the most common liver disorder in western countries, estimated to affect at least one-quarter of the general population and rising up to 90% in morbidly obese individuals [44, 45]. It comprises a disease spectrum ranging from steatosis (fatty liver), through NASH, to fibrosis and ultimately liver cirrhosis. Previous studies mentioned that the pathogenesis of NAFLD commonly can be divided into two hits. The first hit, hepatic triglyceride accumulation (steatosis), increases susceptibility of the liver to injury mediated by second hit, such as inflammation and oxidative stress, which in turn lead to fibrosis [8, 46]. Therefore, finding a proper way which can simultaneously target both of the first and second hits, that is, lipid accumulation, oxidative stress, inflammation, and fibrosis, might be a potential approach to prevent the development of NAFLD in clinics.

In the present study, we provide the first evidence that* magnolia* extract, BL153, attenuated obesity-associated liver damage in a HFD-induced obesity mouse model. Most importantly, BL153 treatment significantly attenuated obesity caused liver pathological changes, including hepatic hypertrophy, lipid accumulation, fibrosis, inflammation, and oxidative stress.

*Magnolia* has been used as Chinese traditional medicine to treat various disorders [9, 10]. It is reported that magnolol, a main compound isolated from* Magnolia* bark, reduces the number of intracellular stored lipid droplets by enhancing lipolysis and thus inhibits the formation of intracellular cholesterol esters [47]. It is unclear whether the liver weight lowering and anti-inflammation effects of BL153 are attributed to scavenging of lipid accumulation in liver. In our study, we examined the hepatic lipid accumulation in both HFD-fed and standard diet-fed mice by Oil Red O staining and triglyceride assay (Figure 3). The result showed that the fat droplets were obviously observed in the liver of HFD-fed mice associated with hepatic necrosis, which were significantly inhibited by BL153 treatment (Figure 3). The current study revealed that lowering hepatic lipid accumulation was the key mechanism of BL153 to fight against the first hit of NAFLD.

Liver weight increase is a main feature of hepatic hypertrophy associated with hepatic fibrosis, which is a key step during the development of NAFLD [48, 49]. Growing evidence demonstrated that* Magnolia* also showed great beneficial effect on antifibrosis. Administration of* Magnolia* cannot only prevent the cardiac fibrosis induced by ischemia/reperfusion but also the renal fibrosis induced by TGF-*β*1 [21, 50]. In the present study, we further confirmed that HFD significantly upregulated hepatic CTGF and TGF-*β*1 expression, which were remarkably attenuated by BL153 treatment in a dose-dependent manner (Figure 2).

As we know, inflammation and the associated oxidative stress also participate in the development of NAFLD. Moreover, other studies also mentioned that another constituent of* Magnolia*, honokiol, plays inhibiting role on lipid accumulation-induced inflammation and oxidative stress [13–15, 51]. Therefore, we tried to determine whether anti-inflammation and antioxidation are the missing mechanisms of BL153 on preventing pathological process of HFD-induced liver damage. Our results indicated that HFD significantly upregulated the expression of hepatic inflammatory factors including TNF-*α*, ICAM-1, and PAI-1 (Figure 4) as well as the marker of oxidative stress including 3-NT and 4-HNE (Figure 5). Administration of BL153 significantly attenuated oxidative stress shown by decrease of 3-NT and 4-HNE expressions in the liver with a dose-dependent manner (Figure 5). BL153 also remarkably inhibited hepatic inflammatory factor expressions without significant differences among three dose level treatments (Figure 4), which implied that BL153 had different sensitivity to HFD-induced inflammation and oxidative stress.

In summary, the fat liver damage is a main consequence of the development of obesity induced by HFD. The* magnolia* extract BL153 can simultaneously induce beneficial effects on HFD-induced liver damage by inhibiting hepatic lipid accumulation, inflammation, oxidative stress, hypertrophy, and fibrosis.
